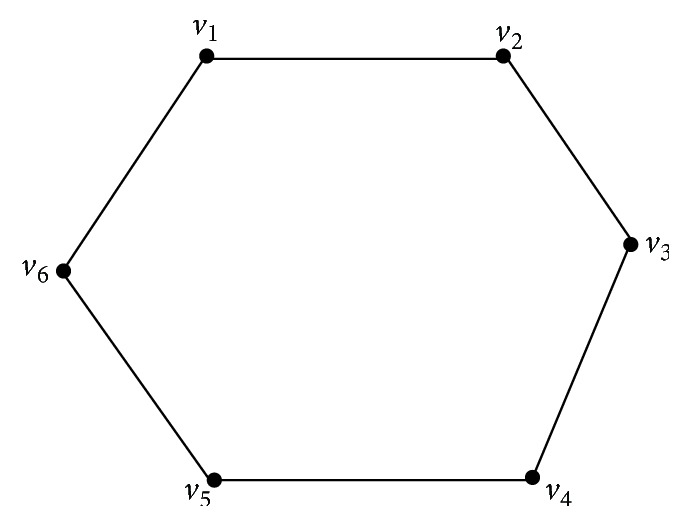# On Vertex Covering Transversal Domination Number of Regular Graphs

**DOI:** 10.1155/2016/1029024

**Published:** 2016-03-29

**Authors:** R. Vasanthi, K. Subramanian

**Affiliations:** ^1^Department of Mathematics, Alagappa Chettiar College of Engineering and Technology, Karaikudi, Tamil Nadu 630 004, India; ^2^Department of Mathematics, Alagappa Government Arts College, Karaikudi, Tamil Nadu 630 003, India

## Abstract

A simple graph *G* = (*V*, *E*) is said to be *r*-regular if each vertex of *G* is of degree *r*. The vertex covering transversal domination number *γ*_vct_(*G*) is the minimum cardinality among all vertex covering transversal dominating sets of *G*. In this paper, we analyse this parameter on different kinds of regular graphs especially for *Q*_*n*_ and *H*_3,*n*_. Also we provide an upper bound for *γ*_vct_ of a connected cubic graph of order *n* ≥ 8. Then we try to provide a more stronger relationship between *γ* and *γ*_vct_.

## 1. Introduction

Hamid [1] introduced independent transversal domination in graphs. It was defined using maximum independent set in a graph. Vasanthi and Subramanian [2] introduced vertex covering transversal domination in graphs using minimum vertex covering set in a graph. The vertex covering transversal domination number of some standard graphs such as *K*_*n*_, *K*_*m*,*n*_, *P*_*n*_, *C*_*n*_, and *W*_*n*_ and trees is dealt with in paper [2]. Bounds of *γ*_vct_ are also established through various parameters in [2]. Lam et al. [3] worked on independent domination number of regular graphs. In this paper, we investigate our parameter *γ*_vct_ for regular graphs. Also we try to provide a more stronger relationship between *γ* and *γ*_vct_.

A simple graph *G* = (*V*, *E*) is said to be *r-regular* if each vertex of *G* is of degree *r*. A set *I*⊆*V* of vertices in *G* is called an* independent set* if no two vertices in *I* are adjacent. Also *I* is said to be a* maximum independent set* if there is no other independent set *I*′ such that |*I*′| > |*I*|. The cardinality of a maximum independent set is called the* independence number* and is denoted by *β*_0_(*G*). A set *C*⊆*V* of vertices in *G* is called a* vertex covering set* (or simply* covering set*) if every edge of *G* is incident to at least one vertex in *C*. Also *C* is said to be a* minimum vertex covering set* if there is no other vertex covering set *C*′ such that |*C*′| < |*C*|. The cardinality of a minimum vertex covering set is called the* vertex covering number* and is denoted by *α*_0_(*G*). A set *D*⊆*V* of vertices in the graph *G* is called a* dominating set* if every vertex in *V* − *D* is adjacent to a vertex in *D*. A dominating set which intersects every minimum vertex covering set in *G* is called a* vertex covering transversal dominating set*. The minimum cardinality of a vertex covering transversal dominating set is called* vertex covering transversal domination number* of *G* and is denoted by *γ*_vct_(*G*).

The parameter* independent domination number i*(*G*) was introduced by Cockanye and Hedetniemi in [4]. The* independent domination number i*(*G*) is the minimum cardinality among all independent dominating sets of *G*. An independent set is dominating if and only if it is maximal. So *i*(*G*) is the minimum cardinality of a maximal independent set in *G*. In paper [3], the following theorem which gives the upper bound for independent domination number of a connected cubic graph has been proved.

**Theorem 1 .**

If *G* is a connected cubic graph of order *n* where *n* ≥ 8, then *i*(*G*) ≤ 2*n*/5.

## 2. Notations

We use the following notations throughout the paper:

  
*α*_0_-set to denote minimum vertex covering set,
  
*β*_0_-set to denote maximum independent set,
  
*γ*-set to denote a dominating set of minimum cardinality,
  
*γ*_vct_-set to denote a vertex covering transversal dominating set of minimum cardinality,
  
*γ*(*G*) to denote domination number of *G*,
  
*γ*_vct_(*G*) to denote vertex covering transversal domination number of *G*,
  
*i*(*G*) to denote independent domination number of *G*,
  
*O*(*G*) to denote the order of *G*,
  deg_*G*_(*u*) to denote the degree of a vertex *u* in *G*.

## 3. *γ*_vct_ for Regular Graphs

Here, we provide the vertex covering transversal domination number of some standard regular graphs such as complete graphs, complete bipartite regular graphs, cycles, and hypercube *Q*_*n*_. We also establish *γ*_vct_ for certain family of regular graphs defined in [3].

**Example 2 .**

*K*_*n*_ is a (*n* − 1)-regular graph and *γ*_vct_(*K*_*n*_) = 2 for *n* ≥ 2.

**Example 3 .**

*C*_*n*_ is a 2-regular graph of order *n* ≥ 3 and

(1) $$\begin{array}{c} \gamma_{\text{vct}}\left(\;C_{n}\;\right)=\left\{\begin{array}{cc} 2 & \text{if }n=3,4\\ 3 & \text{if }n=5\\ \left\lceil \frac{n}{3}\right\rceil & \text{otherwise}. \end{array}\right. \end{array}$$

**Example 4 .**

*K*_*n*,*n*_ is a complete bipartite *n*-regular graph and *γ*_vct_(*K*_*n*,*n*_) = 2.

The following theorem provides the vertex covering transversal domination number of *n*-dimensional cube or hypercube *Q*_*n*_ defined in [5].

**Theorem 5 .**

If *Q*_*n*_ is a hypercube containing 2^*n*^ vertices which is *n*-regular, then

(2) $$\begin{array}{c} \gamma_{vct}\left(\;Q_{n}\;\right)=\left\{\begin{array}{cc} 2 & if\;n=2\\ 2^{n-2} & if\;n\geq 3. \end{array}\right. \end{array}$$

**Proof**

The *n*-dimensional cube or hypercube *Q*_*n*_ contains 2^*n*^ vertices and is *n*-regular. Each vertex in *Q*_*n*_ is represented by a *n*-tuple with 0's and 1's. Two vertices in *Q*_*n*_ are adjacent if and only if the *n*-tuples differ in exactly one position. Also any *v* ∈ *Q*_*n*_ is the *n*-tuple binary number and its complement *v* ^*c*^ is also an *n*-tuple binary number obtained by replacing 0 by 1 and 1 by 0 in *v*. The weight of a 0,1 vertex is the number of 1's occurring in it. There are exactly 2^*n*−1^ vertices of odd weight and 2^*n*−1^ vertices of even weight. Each edge of *Q*_*n*_ consists of a vertex of even weight and a vertex of odd weight. The vertices of even weight form an independent set and so do the vertices of odd weight. Therefore *Q*_*n*_ is bipartite with bipartitions *S*_1_ and *S*_2_ where *S*_1_ is the set of all *n*-tuples of even weight and *S*_2_ is the set of all *n*-tuples of odd weight with |*S*_1_| = |*S*_2_| = 2^*n*−1^.

Also *S*_1_ and *S*_2_ are the only *β*_0_-sets of *Q*_*n*_. Since they are complements of each other, *S*_1_ and *S*_2_ are the only *α*_0_-sets of *Q*_*n*_.

**Figure 1:**
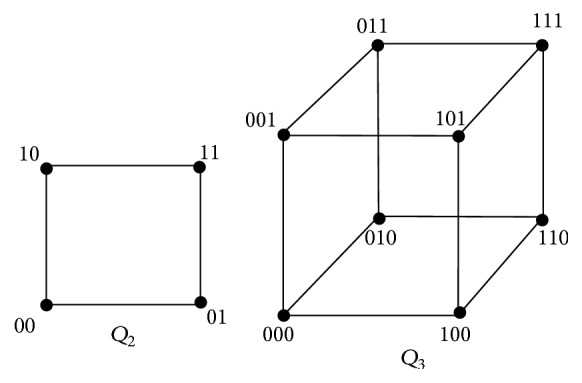


For *n* = 2, *Q*_2_ is as shown in Figure 1. Obviously, *S*_1_ = {00,11} and *S*_2_ = {10,01} are *α*_0_-sets of *Q*_2_. Then *D* = {00,10} is a *γ*_vct_-set of *Q*_2_ and so *γ*_vct_(*Q*_2_) = 2.

Now suppose *n* ≥ 3.

For *n* = 3, *Q*_3_ is the hypercube on 8 vertices which is 3-regular and is represented as in Figure 1. The only two *α*_0_- sets of *Q*_3_ are *S*_1_ = {000,011,110,101} and *S*_2_ = {001,010,100,111}. Then *γ*_vct_(*Q*_3_) = 2 since every two-element set of the form {*v*, *v* ^*c*^} where *v* ∈ *S*_1_ and *v* ^*c*^ ∈ *S*_2_ is a *γ*_vct_-set.

**Figure 2:**
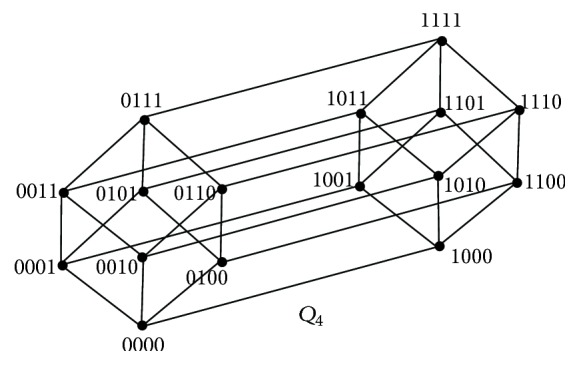


If *n* = 4, the hypercube *Q*_4_ contains 2^4^ vertices and is 4-regular as shown in Figure 2. *Q*_4_ is bipartite with bipartitions *S*_1_ = {0000,0011,0110,1100,0101,1010,1001,1111} and *S*_2_ = {0001,0010,0100,1000,0111,1110,1011,1101}. Also *S*_1_ and *S*_2_ are the only *α*_0_-sets of *Q*_4_.

Let *D* = {0000,1111,0001,1110}. Then *D* is a dominating set of *Q*_4_. Clearly it intersects both *S*_1_ and *S*_2_. Therefore *D* is a vertex covering transversal dominating set and so *γ*_vct_(*Q*_4_) = 4. Hence it remains to show that *D* is of minimum cardinality.

Suppose there exists a vertex covering transversal dominating set *D*′ of cardinality less than 4. It must have at least 2 vertices as it intersects both *S*_1_ and *S*_2_. Suppose |*D*′| = 3. Since each vertex is of degree 4, all the three vertices in *D*′ may dominate at most 12 vertices. But there are 16 vertices in *Q*_4_ and so *D*′ do not dominate at least 1 vertex. This is a contradiction to the assumption that *D*′ is a vertex covering transversal dominating set.

Also any set containing two mutually complementary vertices from *S*_1_, say, 1100, 0011, and the other two mutually complementary vertices from *S*_2_, say, 1000, 0111, form a *γ*_vct_-set. Thus *S* = {*v*_1_, *v*_1_^*c*^, *v*_2_, *v*_2_^*c*^} where *v*_1_, *v*_1_^*c*^ ∈ *S*_1_ and *v*_2_, *v*_2_^*c*^ ∈ *S*_2_ is a *γ*_vct_-set of *Q*_4_.

If *n* = 5, the hypercube *Q*_5_ contains 2^5^ = 32 vertices and the bipartition *S*_1_ contains 2^4^ vertices and *S*_2_ contains 2^4^ vertices. Let *S* = {*v*_1_, *v*_1_^*c*^, *v*_2_, *v*_2_^*c*^, *v*_3_, *v*_3_^*c*^, *v*_4_, *v*_4_^*c*^} where *v*_1_, *v*_2_, *v*_3_, *v*_4_ ∈ *S*_1_ and *v*_1_^*c*^, *v*_2_^*c*^, *v*_3_^*c*^, *v*_4_^*c*^ ∈ *S*_2_. Then *S* is a *γ*-set which intersects both *S*_1_ and *S*_2_. Hence *γ*_vct_(*Q*_5_) = 2^3^.

Thus in general, *S* = {*v*_1_, *v*_1_^*c*^, *v*_2_, *v*_2_^*c*^,…, *v*_2^*n*−3^−1_, *v*_2^*n*−3^−1_^*c*^, *v*_2^*n*−3^_, *v*_2^*n*−3^_^*c*^} is a *γ*_vct_-set of *Q*_*n*_. In particular, if *n* is odd, *v*_1_, *v*_2_,…, *v*_2^*n*−3^_ ∈ *S*_1_ and *v*_1_^*c*^, *v*_2_^*c*^,…, *v*_2^*n*−3^_^*c*^ ∈ *S*_2_. If *n* is even, *v*_1_, *v*_1_^*c*^, *v*_2_, *v*_2_^*c*^,…, *v*_2^*n*−4^_, *v*_2^*n*−4^_^*c*^ ∈ *S*_1_ and *v*_2^*n*−4^+1_, *v*_2^*n*−4^+1_^*c*^,…, *v*_2^*n*−3^_, *v*_2^*n*−3^_^*c*^ ∈ *S*_2_. Hence *γ*_vct_(*Q*_*n*_) = 2^*n*−2^.

**Theorem 6 .**

If *G* is a connected regular graph of degree *n* − 2 and *O*(*G*) = *n*, then *γ*_*vct*_(*G*) = 2.

**Proof**

Choose any vertex *u* ∈ *V*(*G*). Then deg_*G*_(*u*) = *n* − 2; that is, *u* is adjacent to *n* − 2 vertices in *G*. Then there remains exactly one vertex, say, *v*, which is not adjacent to *u*. Therefore *S* = {*u*, *v*} is an independent set of *G*. Also *v* is adjacent to *n* − 2 vertices in *G* except *u*. Hence no other vertex may be included in *S*. Therefore *S* is a maximum independent set of *G*.

Now let *w* ∈ *V* − *S*. Then *w* is adjacent to both *u* and *v*. Since *u* dominates every vertex in *G* except *v*, and *w* dominates *n* − 2 vertices including *v*, it is obvious that *D* = {*u*, *w*} is a dominating set which intersects every minimum vertex covering set of *G*. Also *D* is of minimum cardinality in *G*. Hence *γ*_vct_(*G*) = 2.

It is noted that {*v*, *w*} is also a *γ*_vct_-set.

**Remark 7 .**

In the above theorem, *n* should be even. For otherwise, if *n* is odd, then *n* − 2 is odd which is impossible as the number of vertices of odd degree in a graph is even.

**Lemma 8 .**

Given positive integers *p* ≥ 2 and *q* ≥ 3, let *G*(*p*, *q*) be the family of graphs such that *V* = ⋃_*i*=1_^*p*^(*X*_*i*_ ∪ *Y*_*i*_ ∪ *Z*_*i*_) and *E* = *E*_1_ ∪ *E*_2_ ∪ *E*_3_ ∪ *E*_4_ with

  
*X*_*i*_ = {*x*_*i*1_, *x*_*i*2_,…, *x*_*i*(*q* − 1)_}, *Y*_*i*_ = {*y*_*i*1_, *y*_*i*2_,…, *y*_*iq*_}, *Z*_*i*_ = {*z*_*i*1_, *z*_*i*2_,…, *z*_*iq*_},
  
*E*_1_ = ⋃_*i*=1_^*p*^{*x*_*ik*_ *y*_*il*_; 1 ≤ *k* ≤ *q* − 1, 1 ≤ *l* ≤ *q*},
  
*E*_2_ = ⋃_*i*=1_^*p*^{*y*_*ik*_ *z*_*ik*_; 1 ≤ *k* ≤ *q*},
  
*E*_3_ = ⋃_*i*=1_^*p*^{*z*_*ik*_ *z*_*il*_; 1 ≤ *k*, *l* ≤ *q*, *k* ≠ *l*} − {*z*_*i*1_ *z*_*iq*_},
  
*E*_4_ = {*z*_*iq*_ *z*_(*i* + 1)1_; 1 ≤ *i* ≤ *p* − 1} ∪ {*z*_*pq*_ *z*_11_}.

Then

(i) |*V*(*G*)| = *p*(3*q* − 1),
(ii) *G*(*p*, *q*) is connected and *q*-regular,
(iii) *γ*_*vct*_(*G*(*p*, *q*)) = 4*p*.

**Proof**

*G*(*p*, *q*) contains *p* subgraphs which we shall call blocks each containing 3*q* − 1 vertices and isomorphic to each other. By the edge set *E*_4_, we observe that they are connected to each other.

Thus (i) and (ii) are obvious.

**Figure 3:**
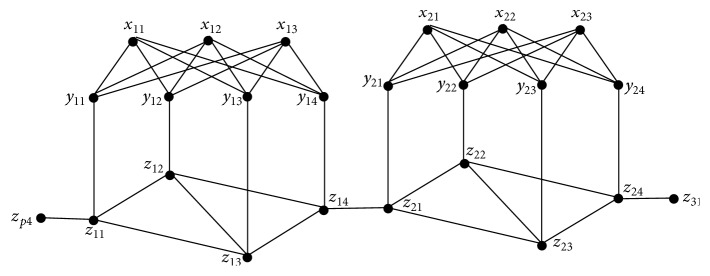


For *q* = 4, two connected blocks of *G*(*p*, 4) each consisting of 11 vertices are as shown in Figure 3.

Now *I* = ⋃_*i*=1_^*p*^ *Y*_*i*_ is a maximum independent set of *G*(*p*, *q*). Then its complement *S* = *V* − *I* = ⋃_*i*=1_^*p*^(*X*_*i*_ ∪ *Z*_*i*_) is a minimum vertex covering set of *G*(*p*, *q*).

Also *I*_*j*_ = ⋃_*i*=1_^*p*^(*Y*_*i*_ − {*y*_*ij*_}) ∪ {*z*_*ij*_} and *J*_*j*_ = ⋃_*i*=1_^*p*^(*X*_*i*_) ∪ {*z*_*ij*_}, 1 ≤ *j* ≤ *q* are maximum independent sets in *G*(*p*, *q*). Let *S*_*j*_ and *T*_*j*_ be the complement of each *I*_*j*_ and *J*_*j*_. Then *S*_*j*_ = ⋃_*i*=1_^*p*^((*X*_*i*_ ∪ *Z*_*i*_) − {*z*_*ij*_} ∪ {*y*_*ij*_}) and *T*_*j*_ = ⋃_*i*=1_^*p*^((*Y*_*i*_ ∪ *Z*_*i*_) − {*z*_*ij*_}), 1 ≤ *j* ≤ *q* are minimum vertex covering sets in *G*(*p*, *q*). Now the subgraph *G*_*i*_ induced by *X*_*i*_ ∪ *Y*_*i*_ in each block is a complete bipartite graph *K*_*q*−1,*q*_.

Since *γ*_vct_(*K*_*m*,*n*_) = 2 if *m*, *n* > 1, we have *γ*_vct_(*K*_*q*−1,*q*_) = 2. Also each {*x*_*ij*_, *y*_*ik*_} for 1 ≤ *j* ≤ *q* − 1 and 1 ≤ *k* ≤ *q* is a vertex covering transversal dominating set of *G*_*i*_. Then {*x*_*ij*_, *y*_*ik*_, *z*_*i*1_, *z*_*iq*_}, 1 ≤ *j* ≤ *q* − 1 and 1 ≤ *k* ≤ *q*, is a dominating set for each block. Therefore *D*_*jk*_ = ⋃_*i*=1_^*p*^{*x*_*ij*_, *y*_*ik*_, *z*_*i*1_, *z*_*iq*_}, 1 ≤ *j* ≤ *q* − 1 and 1 ≤ *k* ≤ *q*, is a *γ*-set which intersects the only *α*_0_-sets *S*, *S*_*j*_ and *T*_*j*_ of *G*(*p*, *q*) for each *j*.

Hence *γ*_vct_(*G*(*p*, *q*)) = 4*p*.

**Theorem 9 .**

If *q* ≥ 3, then there exists a connected *q*-regular graph with *γ*_*vct*_(*G*)≥⌈4*n*/3*q*⌉ where *n* is the order of *G*.

**Proof**

Let *G* = *G*(*p*, *q*) be defined as in Lemma 8. Then *γ*_vct_(*G*)/*n* = 4/(3*q* − 1) ≥ 4/3*q*. Thus *γ*_vct_(*G*) ≥ 4*n*/3*q*.

**Lemma 10 .**

Given positive integers *p* ≥ 1 and *q* ≥ 2, let *G* ^*∗*^(*p*, *q*) be the graph (*V*, *E*) with *V* = *U* ∪ [⋃_*i*=1_^2*p*+1^(*V*_*i*_ ∪ *W*_*i*_)] and *E* = *E*_1_ ∪ *E*_2_ ∪ *E*_3_ ∪ *E*_4_ with

  
*U* = {*u*_1_, *u*_2_,…, *u*_2*p*+1_}, *V*_*i*_ = {*v*_*i*1_, *v*_*i*2_,…, *v*_*i*(*q* + 2*p*)_}, *W*_*i*_ = {*w*_*i*1_, *w*_*i*2_,…, *w*_*i*(*q* + 2*p* − 1)_},
  
*E*_1_ = {*u*_*j*_ *u*_*k*_; 1 ≤ *j* < *k* ≤ 2*p* + 1},
  
*E*_2_ = ⋃_*i*=1_^2*p*+1^{*u*_*i*_ *v*_*ik*_; 1 ≤ *k* ≤ *q*},
  
*E*_3_ = ⋃_*i*=1_^2*p*+1^{*v*_*i*(*q*+2*k*−1)_ *v*_*i*(*q*+2*k*)_; 1 ≤ *k* ≤ *p*},
  
*E*_4_ = ⋃_*i*=1_^2*p*+1^{*v*_*ik*_ *w*_*il*_; 1 ≤ *k* ≤ *q* + 2*p*, 1 ≤ *l* ≤ *q* + 2*p* − 1}.

Then

(i) |*V*(*G*)| = 2(2*p* + 1)(*q* + 2*p*),
(ii) *G* ^*∗*^(*p*, *q*) is connected and (*q* + 2*p*)-regular,
(iii) *γ*_*vct*_(*G* ^*∗*^(*p*, *q*)) = 2(2*p* + 1).

**Proof**

**Figure 4:**
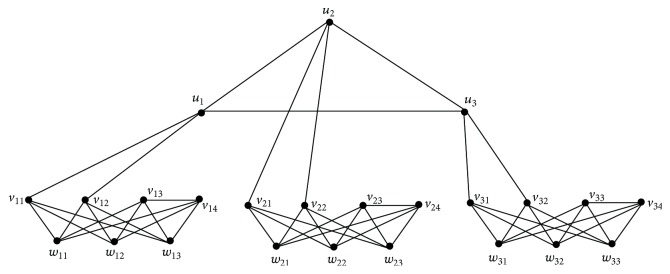


(i) and (ii) are obvious. If *p* = 1 and *q* = 2, the graph *G* ^*∗*^(1,2) is as shown in Figure 4.

It is clear that each *I*_*j*_ = [⋃_*i*=1_^2*p*+1^(*W*_*i*_)] ∪ {*u*_*j*_}, *j* = 1 to 2*p* + 1, is a maximum independent set in *G* ^*∗*^(*p*, *q*). Therefore its complement *J*_*j*_ = *V* − *I*_*j*_ = [⋃_*i*=1_^2*p*+1^(*V*_*i*_)]∪[*U* − {*u*_*j*_}], *j* = 1 to 2*p* + 1, is an *α*_0_ set in *G* ^*∗*^(*p*, *q*). Further *J*_1_, *J*_2_,…, *J*_2*p*_ and *J*_2*p*+1_ are the only *α*_0_-sets of *G* ^*∗*^(*p*, *q*). Now each *S*_*jk*_ = ⋃_*i*=1_^2*p*+1^{*v*_*ik*_, *w*_*ij*_}, *j* = 1 to *q* + 2*p* − 1, *k* = 1 to *q* is a dominating set intersecting *J*_1_, *J*_2_,…, *J*_2*p*_ and *J*_2*p*+1_ and also of minimum cardinality 2(2*p* + 1).

Hence *γ*_vct_(*G* ^*∗*^(*p*, *q*)) = 2(2*p* + 1).

**Theorem 11 .**

For every *r* ≥ 4, there exists a connected *r*-regular graph *G* of order *n* such that *γ*_*vct*_(*G*) = *n*/*r*.

**Proof**

Let *G* = *G* ^*∗*^(*p*, *q*) be defined as in Lemma 10. Then *G* is a connected *r*-regular graph with *r* = *q* + 2*p*. Also *γ*_vct_(*G*)/*n* = 1/(*q* + 2*p*).

Thus *γ*_vct_(*G*) = *n*/*r*.

**Remark 12 .**

Theorems 9 and 11 hold good if *γ*_vct_ is replaced by *γ*.

## 4. *γ*_vct_ for Regular Cubic Graphs

In this section, we provide the vertex covering transversal domination number of some regular cubic graphs especially Harary graph *H*_3,*n*_ defined in [6]. We also obtain an upper bound for the vertex covering transversal domination number of a connected cubic graph.

**Example 13 .**

**Figure 5:**
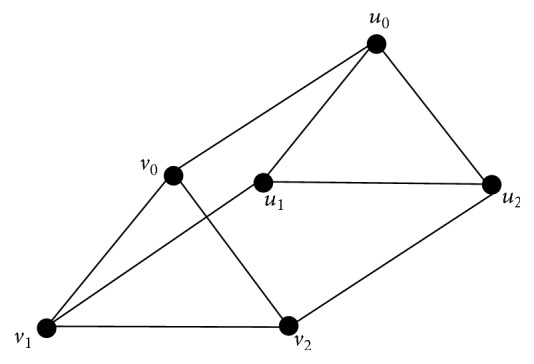


Consider the triangular prism graph *Y*_3_ shown in Figure 5. It is a regular cubic graph.

*Y*_3_ has 6 vertices and 9 edges. Assume that the graph *Y*_3_ is labelled as shown in the diagram. It is clear that {*u*_*i*mod⁡3_, *v*_(*i* + 1)mod⁡3_} and {*u*_*i*mod⁡3_, *v*_(*i* + 2)mod⁡3_} for *i* = 0,1, 2 are *β*_0_-sets of *Y*_3_. Then their complements *C*_*i*_ = {*u*_(*i* + 1)mod⁡3_, *u*_(*i* + 2)mod⁡3_, *v*_*i*mod⁡3_, *v*_(*i* + 2)mod⁡3_} and *S*_*i*_ = {*u*_(*i* + 1)mod⁡3_, *u*_(*i* + 2)mod⁡3_, *v*_*i*mod⁡3_, *v*_(*i* + 1)mod⁡3_} for *i* = 0,1, 2 are *α*_0_-sets of *Y*_3_. Now each *D*_*i*_ = {*u*_*i*mod⁡3_, *v*_*i*mod⁡3_}, *i* = 0,1, 2 is a *γ*-set for *Y*_3_. Clearly it intersects each *C*_*i*_ and *S*_*i*_. Therefore *γ*_vct_(*Y*_3_) = 2.

**Example 14 .**

**Figure 6:**
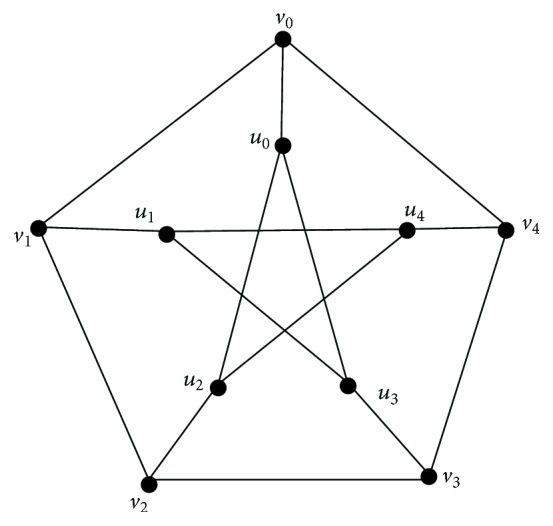


Consider Peterson graph which is cubic regular shown in Figure 6.

Assuming that the graph *G* is labelled as shown in Figure 6, it is obvious that *I*_*i*_ = {*v*_*i*mod⁡5_, *v*_(*i* + 3)mod⁡5_, *u*_(*i* + 1)mod⁡5_, *u*_(*i* + 2)mod⁡5_}, *i* = 0,1, 2,3, 4 are *β*_0_-sets of *G*. Then their complements *C*_*i*_ = {*v*_(*i* + 1)mod⁡5_, *v*_(*i* + 2)mod⁡5_, *v*_(*i* + 4)mod⁡5_, *u*_*i*mod⁡5_, *u*_(*i* + 3)mod⁡5_, *u*_(*i* + 4)mod⁡5_}, *i* = 0,1, 2,3, 4 are *α*_0_-sets of *G*. Now *S*_*i*_ = {*v*_*i*mod⁡5_, *u*_(*i* + 2)mod⁡5_, *u*_(*i* + 3)mod⁡5_} are *γ*-sets intersecting each *C*_*i*_. Hence *γ*_vct_(*G*) = 3.

Note that *S*_*i*_ = {*v*_*i*mod⁡5_, *v*_(*i* + 3)mod⁡5_, *u*_(*i* + 4)mod⁡5_} are also *γ*_vct_-sets in *G*.

**Theorem 15 .**

If *H*_3,*n*_ is a Harary graph with *n* ≥ 6, then *γ*_*vct*_(*H*_3,*n*_) = ⌊(*n* + 1)/3⌋.

**Proof**

*H*_3,*n*_ is a 3-regular graph and so *n* is even. By the definition of *H*_3,*n*_, every vertex *v*_*i*_ ∈ *H*_3,*n*_ is adjacent to the vertices *v*_*i*+1_, *v*_*i*−1_, and *v*_*i*+*k*_ where *n* = 2*k*.

**Figure 7:**
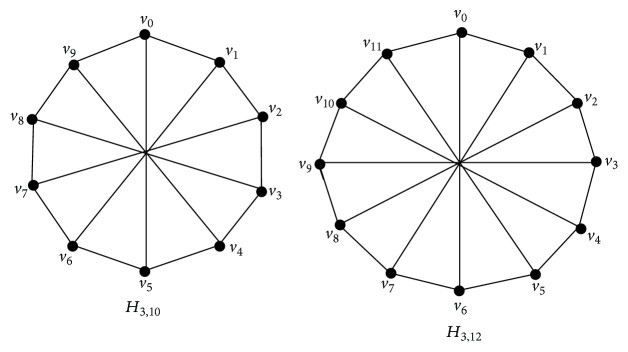


Let *V*(*H*_3,*n*_) = {*v*_0_, *v*_1_, *v*_2_,…, *v*_*n*−1_}. The graphs *H*_3,10_ and *H*_3,12_ are shown in Figure 7.

*Case  1*. Suppose *n* = 2*k* where *k* is odd.

Then *C*_1_ = {*v*_0_, *v*_2_, *v*_4_,…, *v*_*n*−2_} and *C*_2_ = {*v*_1_, *v*_3_, *v*_5_,…, *v*_*n*−1_} are the only *α*_0_-sets of *H*_3,*n*_.

*Subcase  1*. Let *n* ≡ 0 (mod⁡3).

Then *S* = {*v*_0_, *v*_3_, *v*_6_,…, *v*_*n*−3_} is a *γ*-set which intersects *C*_1_ and *C*_2_ and |*S*| = *n*/3.

*Subcase  2*. Suppose *n* ≡ 1 (mod⁡3).

Then *S* = {*v*_0_, *v*_3_, *v*_6_,…, *v*_*n*−4_} is a *γ*-set which intersects *C*_1_ and *C*_2_ and |*S*| = (*n* − 1)/3.

*Subcase  3*. Suppose *n* ≡ 2 (mod⁡3).

Then *S* = {*v*_0_, *v*_3_, *v*_6_,…, *v*_*n*−2_} is a *γ*-set which intersects *C*_1_ and *C*_2_ with |*S*| = (*n* + 1)/3.

Thus in all the subcases of Case  1, *γ*_vct_(*H*_3,*n*_) = ⌊(*n* + 1)/3⌋.

*Case  2*. Suppose *n* = 2*k* where *k* is even.

Then *I*_*i*_ = {*v*_*i*mod⁡*n*_, *v*_(*i* + 2)mod⁡*n*_,…, *v*_(*i* + *k* − 2)mod⁡*n*_, *v*_(*i* + *k* + 1)mod⁡*n*_, *v*_(*i* + *k* + 3)mod⁡*n*_,…, *v*_(*i* + *n* − 5)mod⁡*n*_, *v*_(*i* + *n* − 3)mod⁡*n*_} is a *β*_0_-set for each *i* = 0,1, 2,…, *n* − 1.

Therefore *C*_*i*_ = {*v*_(*i* + 1)mod⁡*n*_, *v*_(*i* + 3)mod⁡*n*_,…, *v*_(*i* + *k* − 1)mod⁡*n*_, *v*_(*i* + *k*)mod⁡*n*_, *v*_(*i* + *k* + 2)mod⁡*n*_,…, *v*_(*i* + *n* − 4)mod⁡*n*_, *v*_(*i* + *n* − 2)mod⁡*n*_, *v*_(*i* + *n* − 1)mod⁡*n*_} is an *α*_0_-set for each *i* = 0,1, 2,…, *n* − 1.

*Subcase  1*. Let *n* ≡ 0 (mod⁡3).

Then *S*_*i*_ = {*v*_*i*mod⁡*n*_, *v*_(*i* + 3)mod⁡*n*_, *v*_(*i* + 6)mod⁡*n*_,…, *v*_(*i* + *n* − 3)mod⁡*n*_} is a *γ*-set which intersects each *C*_*i*_ for *i* = 0,1, 2,…, *n* − 1.

*Subcase  2*. Let *n* ≡ 1 (mod⁡3).

Then *S*_*i*_ = {*v*_*i*mod⁡*n*_, *v*_(*i* + 3)mod⁡*n*_, *v*_(*i* + 6)mod⁡*n*_,…, *v*_(*i* + *n* − 4)mod⁡*n*_} is a *γ*-set which intersects each *C*_*i*_ for *i* = 0,1, 2,…, *n* − 1.

*Subcase  3*. Let *n* ≡ 2 (mod⁡3).

Then *S*_*i*_ = {*v*_*i*mod⁡*n*_, *v*_(*i* + 3)mod⁡*n*_, *v*_(*i* + 6)mod⁡*n*_,…, *v*_(*i* + *n* − 2)mod⁡*n*_} is a *γ*-set which intersects each *C*_*i*_ for *i* = 0,1, 2,…, *n* − 1.

The *γ*_vct_-sets mentioned in all the subcases of Case  2 are also of cardinality ⌊(*n* + 1)/3⌋.

Thus *γ*_vct_(*H*_3,*n*_) = ⌊(*n* + 1)/3⌋.

**Remark 16 .**

In most of the graphs considered by us, it is observed that *γ*_vct_ = *γ*.

**Theorem 17 .**

If *G* is a connected cubic graph of order *n* with *n* ≥ 8, then *γ*_*vct*_(*G*)≤⌈2*n*/5⌉.

**Proof**

Let *I* be an independent dominating set of cardinality *i*(*G*). Then *I* is a maximal independent set of minimum cardinality. Since *I* is independent, no two vertices of *I* are adjacent in *G*. Let *J* = *V* − *I*. Then the vertices in *I* are adjacent only to the vertices in *J*.

*Case  1*. Suppose *I* itself is a *β*_0_-set. Then *J* is an *α*_0_-set. Let *S* = *I* ∪ {*v*} where *v* ∈ *J*. Then *S* is a vertex covering transversal dominating set of *G*. Therefore *γ*_vct_(*G*) ≤ *i*(*G*) + 1. Hence *γ*_vct_(*G*) ≤ 2*n*/5 + 1 (by Theorem 1 proved in [3]).

*Case  2*. Suppose *I* is not a *β*_0_-set. But *I* is a maximal independent dominating set of minimum cardinality. We claim that *I* intersects every *α*_0_-set of *G*.

Suppose that *I* does not intersect an *α*_0_-set *C* of *G*. Then *I* ⊂ *V* − *C* where *V* − *C* is a *β*_0_-set of *G*. This is a contradiction to the maximality of *I*.

Hence *I* itself is a vertex covering transversal dominating set of *G*. Therefore *γ*_vct_(*G*) ≤ 2*n*/5.

Thus Cases  1 and 2 imply that *γ*_vct_(*G*)≤⌈2*n*/5⌉.

## 5. Relation between *γ*(*G*) and *γ*_vct_(*G*)

In this section, we prove a more stronger relationship between *γ* and *γ*_vct_ than that proved in [2]. In view of the results and theorems dealt with in the previous sections, we try to characterize graphs for which *γ* = *γ*_vct_ and *γ* < *γ*_vct_.

**Theorem 18 .**

If *G* is a simple connected graph, then *γ*_*vct*_(*G*) ≤ *γ*(*G*) + 1.

**Proof**

Let *D* be a minimum dominating set. If *D* = *V*(*G*), then obviously *γ*_vct_(*G*) = *γ*(*G*). If not, then *D* ⊂ *V*(*G*) and *V*(*G*) − *D* ≠ *ϕ*. Let *u* ∈ *V*(*G*) − *D*. Then *u* is dominated by some vertex *v* in *D*. Let *S* = *D* ∪ {*u*}. Since *uv* is an edge in *G*, either *u* or *v* is included in every minimum vertex covering set of *G*. This implies that *S* intersects every minimum vertex covering set in *G*. Hence *γ*_vct_(*G*) ≤ *γ*(*G*) + 1.

**Theorem 19 .**

Let *G* be a simple connected graph. If there exists a *γ*-set which is not independent, then *γ*_*vct*_(*G*) = *γ*(*G*).

**Proof**

Let *D* be a minimum dominating set which is not an independent set of *G*. Then at least two vertices, say, *u*, *v* in *D*, are adjacent to each other. Therefore *uv* is an edge in *G* and hence either *u* or *v* lies in every minimum vertex covering set of *G*. So *D* intersects every *α*_0_-set of *G*. Therefore *D* itself is a *γ*_vct_-set. Hence *γ*_vct_(*G*) = *γ*(*G*).

**Remark 20 .**

The converse is not true. If *γ*_vct_(*G*) = *γ*(*G*), then there may exist a *γ*-set which is independent also. For example, consider *C*_6_, the cycle on 6 vertices as shown in Figure 8.

Obviously {*v*_1_, *v*_3_, *v*_5_} and {*v*_2_, *v*_4_, *v*_6_} are the only *α*_0_-sets of *C*_6_. Also {*v*_1_, *v*_4_} is a *γ*-set which is independent. Further, it is a *γ*_vct_-set as it intersects both the *α*_0_-sets of *C*_6_. Thus there exists a *γ*-set which is independent in *C*_6_ even though *γ*_vct_(*C*_6_) = *γ*(*C*_6_).

**Remark 21 .**

Now, the obvious question is “If *γ*_vct_(*G*) = *γ*(*G*), is every *γ*-set of *G* a *γ*_vct_-set?” The answer is “not always.” The *γ*-sets and *γ*_vct_-sets in the graphs *Q*_2_ and *Y*_3_ discussed in the previous sections are the best examples for it. So it is noted that this happens if there exists a *γ*-set which is also a *β*_*o*_-set. It obviously produces the result that “If *γ*_vct_(*G*) = *γ*(*G*) = *β*_*o*_(*G*), then there exists at least one *γ*-set in *G* which is not a *γ*_vct_-set.” The next general question is that “What happens if all the *γ*-sets of *G* are *β*_0_-sets?”. The following theorem provides the answer to it.

**Theorem 22 .**

Let *G* be a simple connected graph. If every *γ*-set of *G* is a *β*_0_-set, then *γ*_*vct*_(*G*) = *γ*(*G*) + 1.

**Proof**

Since every *γ*-set *D* of *G* is a *β*_0_-set, choose a vertex *v* in its complement. This is possible since *D* ≠ *V*(*G*) as *D* is a *β*_0_-set of a connected graph *G*. Obviously *D* is not a *γ*_vct_-set as it does not intersect the *α*_0_-set *V*(*G*) − *D*. Let *S* = *D* ∪ {*v*}. We claim that *S* intersects every *α*_0_-set of *G*. Suppose that *S*∩*C* = Φ for some *α*_0_-set *C* in *G*. Then *S*⊆*I* where *I* = *V*(*G*) − *C* is a *β*_0_-set. This implies that *β*_0_(*G*) + 1 ≤ *β*_0_(*G*) which is a contradiction. Hence *S* intersects every *α*_0_-set of *G*. Also *S* is a *γ*_vct_-set of *G* as it contains exactly one vertex more than that of the *γ*-set *D*. Thus *γ*_vct_(*G*) = *γ*(*G*) + 1.

**Remark 23 .**

It is easy to conclude that even though *γ*_vct_ = *γ*, there are graphs in which *γ*-sets do not become *γ*_vct_-sets. This implies that the collection of *γ*_vct_-sets in such graphs is contained in the collection of *γ*-sets. So this may lead to consider *γ*_vct_-sets in the graphs for which *γ*_vct_ = *γ* when we are in a situation to select a minimum number of *γ*-sets in such graphs. This approach may affect a new variation in domination theory.

## Figures and Tables

**Figure 8:**